# Exposure of ELF-EMF and RF-EMF Increase the Rate of Glucose Transport and TCA Cycle in Budding Yeast

**DOI:** 10.3389/fmicb.2016.01378

**Published:** 2016-08-31

**Authors:** Kang-Wei Lin, Chuan-Jun Yang, Hui-Yong Lian, Peng Cai

**Affiliations:** ^1^Physical Environment Group, Key Laboratory of Urban Environment and Health, Institute of Urban Environment, Chinese Academy of SciencesXiamen, China; ^2^College of Resources and Environment, University of the Chinese Academy of SciencesBeijing, China

**Keywords:** ELF-EMF, RF-EMF, TCA, yeast, glucose transportation

## Abstract

In this study, we investigated the transcriptional response to 50 Hz extremely low frequency electromagnetic field (ELF-EMF) and 2.0 GHz radio frequency electromagnetic field (RF-EMF) exposure by Illumina sequencing technology using budding yeast as the model organism. The transcription levels of 28 genes were upregulated and those of four genes were downregulated under ELF-EMF exposure, while the transcription levels of 29 genes were upregulated and those of 24 genes were downregulated under RF-EMF exposure. After validation by reverse transcription quantitative polymerase chain reaction (RT-qPCR), a concordant direction of change both in differential gene expression (DGE) and RT-qPCR was demonstrated for nine genes under ELF-EMF exposure and for 10 genes under RF-EMF exposure. The RT-qPCR results revealed that ELF-EMF and RF-EMF exposure can upregulate the expression of genes involved in glucose transportation and the tricarboxylic acid (TCA) cycle, but not the glycolysis pathway. Energy metabolism is closely related with the cell response to environmental stress including EMF exposure. Our findings may throw light on the mechanism underlying the biological effects of EMF.

## Introduction

Along with the rapid development of electric power and wireless communication equipment utilization, the strength, complexity, and coverage range of extremely low frequency electromagnetic field (ELF-EMF) and radio frequency electromagnetic field (RF-EMF) are increasing. Consequently, concerns regarding the health effects of ELF-EMF and RF-EMF have been raised. The question of whether ELF-EMF and RF-EMF induce biological effects that might be harmful to human health and the environment remains a controversial issue. Since an epidemiological study, conducted in 1979, found that the intensity of ELF-EMF is related to a high risk of childhood leukemia (Wertheimer and Leeper, 1979), the problem of electromagnetic pollution has attracted increasing attention. The International Agency for Research on Cancer (IARC) classified ELF-EMF and RF-EMF as suspected carcinogens (2B) in 2002 and 2011, respectively (IARC, 2002, 2013).

Although many studies have investigated the biological effects of RF-EMF and ELF-EMF at the epidemiological level as well as cellular- and molecular-levels, the basic interactions between these relatively weak fields and living material remain unclear. Numerous hypotheses have been proposed, but none of these is convincingly supported by experimental data. It is generally accepted that the energy introduced by RF-EMF and ELF-EMF is not sufficient to directly break molecular bonds, which makes it difficult to explain the biological effects of EMF. To date, the biological effects of RF-EMF and ELF-EMF still remain controversial, and the EMF research community should pay equal attention to the negative reports as to the positive ones. Therefore, further studies on the biological effects of and the risks posed by RF-EMF and ELF-EMF are of vital importance.

The recent availability of transcriptome sequences in combination with Illumina sequencing technology, which is far more precise and sensitive than other methods for measuring transcript levels (Luan et al., 2011), has provided unprecedented opportunities to investigate the transcriptional response to RF-EMF and ELF-EMF, particularly for discovering the mechanisms underlying the biological effects of EMF. To date, relatively few studies using large-scale screening have analyzed the effects of ELF-EMF and RF-EMF on gene transcription in organisms, and the results have been contradictory. Some researchers have shown that the transcription levels of some genes are affected by EMF exposure (Olivares-Bañuelos et al., 2004; Remondini et al., 2006; Collard et al., 2011), while others have shown that the transcription profile is unchanged by EMF exposure (Luceri et al., 2005; Paparini et al., 2008; Dawe et al., 2009). Only a few investigations on the effects of EMF on yeast cells have been undertaken, even though their short cell cycle, easy handling, extensive characterization, and eukaryotic genetic background make yeast a good model organism for this type of study. One study conducted with budding yeast, showed that no changes in the transcription levels of the tested genes under 0.4-mT 50-Hz ELF-MF, whereas the transcription levels of two genes, *SMC3* and *AQY2 (m)*, were found to be upregulated after exposure to 1800-MHz RF-EMF at a specific absorption rate of 4.7 W/kg for 6 h (Chen et al., 2012). To clarify whether EMF exposure can affect the expression of any gene, and potentially identify genes that are sensitive to EMF exposure, more large-scale screening studies are needed.

In this study, we investigated the transcriptional response under exposure to 50-Hz ELF-EMF and 2.0-GHz RF-EMF exposure by Illumina sequencing technology using budding yeast as the model organism, and we validated the differentially expressed genes (DEGs) by RT-qPCR.

## Methods

### Yeast strains and media

The *S. cerevisiae* strain used in this study was SB34 (*MATa; erg6::TRP1; pDAL5::ADE2; ade2-1; trp1-1; leu2-3,112; his3-11,15; ura2::HIS3, [URE3]*). Yeast were grown on YPD (1% yeast extract, 2% peptone, and 2% D-glucose). The solid medium contained 2% agar.

### Exposure system

The electromagnetic exposure system contained two combined solenoid systems and a biochemical incubator. Each solenoid was 15 cm in length and 40 cm in diameter, and was wrapped with 260 turns of single-strand copper wire. The distance between the closest ends of the two coils was 15 cm. A silicone tube connected to a condenser was wound around the coils to counteract the generated heat. Variable-frequency power sources were connected to the solenoid systems to produce an electromagnetic field, which could be regulated by adjusting the current intensity, frequency, and voltage. The parameters of the exposure system could be regulated within the following ranges: frequency, 40–499.9 Hz; magnetic field, 0.05–7 mT; or electric field, 10–1000 V/m. To detect the stability of the magnetic field generated by this exposure system, measurements were performed using a Portable Field Meter (PMM8053B, Italy). YPD plates containing yeast cells were placed in a space between the two coils. In the sample area (round in shape with a diameter of 24 cm), the difference in the magnetic field strength was less than 5%. The exposure condition used in this study was set to 50 Hz, 6 mT, and the electric field in the sample area was 205 V/m. To prevent the exposure system from influencing the control group, the incubator for the control group was placed in an area where the magnetic field was equal to the background level.

The RF electromagnetic field was generated using a vector signal generator (Agilent E8267D PSG, USA) and signal amplifier (AV38701E, the 41st Institute of CETC, China). The RF-EMF was emitted from an antenna (ETS 3180B), which was placed 24 cm above the sample area. A signal amplifier was used to amplify the RF/MW signal induced by the signal generator. The signal at the sample position was measured using an electromagnetic radiation analyzer (PMM 8053B, Narta-STS, Italy) and a signal analyzer (Agilent N9030A). In this study, yeast cells were exposed to 2000-MHz RF-EMF with a continuous sine wave.

At the position of the yeast cells, the RF electromagnetic field strength was 20 V/m, and the temperature was 30°C. The average specific absorption rate (SAR) for a single cell was 0.12 W/kg. The SAR was calculated using finite difference time-domain (FDTD) analysis methods.

To ensure temperature accuracy and stability, a series of operations was applied. First, the temperature probe of the incubator was put at a position adjacent to the sample so that the incubator would maintain its temperature according to that position. Second, the temperature of the two incubators for the control samples and exposure samples was routinely calibrated using the same thermometer. Third, during the incubation and exposure periods, the temperature of the sample area was continuously monitored using temperature probes placed surrounding the control and exposure samples. Fourth, the surface temperature of each sample during the incubation period was checked using a thermal imager (Testo 890). All of the monitoring data showed that the temperature of the control and exposure samples remained stable.

### Exposure to RF-EMF and ELF-EMF

Yeast cells were incubated in liquid yeast extract peptone dextrose (YPD) medium until they reached the stationary phase. After diluted to 2000 cells per microlitre, 200-μl cells were spread onto YPD plate with an expectation of obtaining around 300–500 colonies on each plate. The plates were then exposed to 2.0-GHz RF-EMF or 50-Hz ELF-EMF at 30°C, whereas control plates were incubated in a control incubator at 30°C.

### RNA isolation

After 96 h of incubation under RF-EMF, ELF-EMF, or control conditions, the yeast cells were harvested by rinsing with 10-ml sterile water and centrifuging at 2500 rpm for 5 min. Total RNA was extracted using a yeast RNA kit (Omega) following the manufacturer's protocol. The RNA integrity was confirmed using a 2200 Bioanalyzer (Agilent Technologies) with a minimum RNA integrated number value of 8.

### DEG library preparation and sequencing

Using the differential gene expression (DGE) method, which generates absolute rather than relative gene expression measurements and avoids many of the inherent limitations of microarray analyses, the gene expression variations were analyzed between the samples exposed to EMF and the control groups. First, mRNA was purified from 6 μg total RNA from each of the samples with magnetic oligo (dT) beads. First- and second-strand cDNA was synthesized, and bead-bound cDNA was subsequently digested with NlaIII, which recognizes CATG sites. The cDNA fragments with 3' ends were then purified with magnetic beads, and Illumina adapter 1 was added to their 5' ends. The junction between Illumina adapter 1 and the CATG site is the recognition site of MmeI, which cuts 17 bp downstream of the CATG site, producing tags with adapter 1. After removing the 3′ fragments with magnetic bead precipitation, Illumina adapter 2 was introduced at the 3′ ends of the tags, resulting in the acquisition of tags with different adapters at each end to form a tag library. After 15 cycles of linear PCR amplification, 85-base strips were purified by 6% Tris-borate-EDTA (TBE)-PAGE gel electrophoresis. These strips were then digested, and the single-chain molecules were fixed on an Illumina chip for sequencing. Each molecule was grown into a single-molecule cluster sequencing template through *in situ* amplification. Four types of fluorescence-labeled nucleotides were added, and sequencing was performed using the sequencing-by-synthesis method. Each tunnel generated millions of raw reads with sequencing lengths of 35 bp.

### Tag annotation and data normalization for determination of gene expression levels

Raw sequences were transformed into clean tags by removing adaptor sequences, low-quality sequences (tags with unknown sequences [“N”]), empty reads (sequences with only adaptor sequence but no tags), too-long or too-short tags, and tags with a copy number of 1 (likely a sequencing error). For annotation, all tags were mapped to reference sequences and allowed no more than 1 nucleotide mismatch. Clean tags mapped to reference sequences from multiple genes were filtered, and the remaining clean tags were designated unambiguous tags. For gene expression analysis, the number of unambiguous clean tags for each gene was calculated and then normalized to the number of transcripts per million tags (TPMs). The gene ontology (GO) classification system was used to determine the possible functions of all tagged genes.

### Analysis of DEGs

A rigorous algorithm was developed to identify DEGs between the EMF-exposed and control (no exposure) yeast cells. The false discovery rate (FDR) was used to determine the *P*-value threshold in multiple tests and analyses. We used an FDR of ≤ 0.001 and an absolute value of log_2_ ratio ≥1 as thresholds to determine the significance of gene expression differences.

### Pathway analysis

Different genes usually cooperate with each other to exercise their biological functions. Pathway-based analysis helps to further our understanding of the biological functions of genes. All DEGs were mapped to terms in the Kyoto Encyclopedia of Genes and Genomes (KEGG) database. We looked for significantly enriched metabolic pathways and signal transduction pathways among the DEGs using the same formula used in the GO analysis, and the *Q*-value threshold was ≤ 0.05.

### RT-qPCR analysis

To confirm the results of the DGE analyses, the expression levels of selected genes were measured using RT-qPCR. cDNA was synthesized using a SYBR PrimeScript reverse transcription-PCR (RT-PCR) kit II (Takara). RT-qPCR was performed on a Roche LightCycle 480 II (Roche) with SYBR Premix Ex Taq II (Takara). Each gene was analyzed in triplicate, and the average threshold cycle (CT) was calculated. The primers used in this study are listed in Table S1 online. The dissociation curves and efficiency analyses demonstrated that all the primers were specifically designed. The relative expression levels were calculated using the 2^−ΔΔCT^ method. As an endogenous control, the expression of β-actin was measured in parallel.

### Statistical analysis

The RT-qPCR data were analyzed using the one-way analysis of variance (ANOVA) least-significant-difference (LSD) method. Statistical analyses were performed using SPSS 20.0 software. The data are presented as the mean values with standard deviations (SD). ^“*”^ and ^“**”^indicate *P* < 0.05 and *P* < 0.01, respectively, compared with the control.

## Results

### Differentially expressed genes (DEGs)

Three yeast cell DEG libraries, namely those of the control group, the RF-EMF exposure group and the ELF-EMF exposure group were sequenced, and approximately 2.5 million raw reads for each sample were generated. After removing adaptor sequences, low-quality reads, and high-N-base reads, the total number of clean reads per library ranged from 2.2 to 2.3 million (from 89.47 to 90.25%). During the process of base calling, the number of clean Q20 reads ranged from 98.27 to 99.2%, whereas the number of clean Q30 reads ranged from 96.68 to 98.36%, demonstrating that we obtained DEG libraries of high quality.

**Figure 1:**
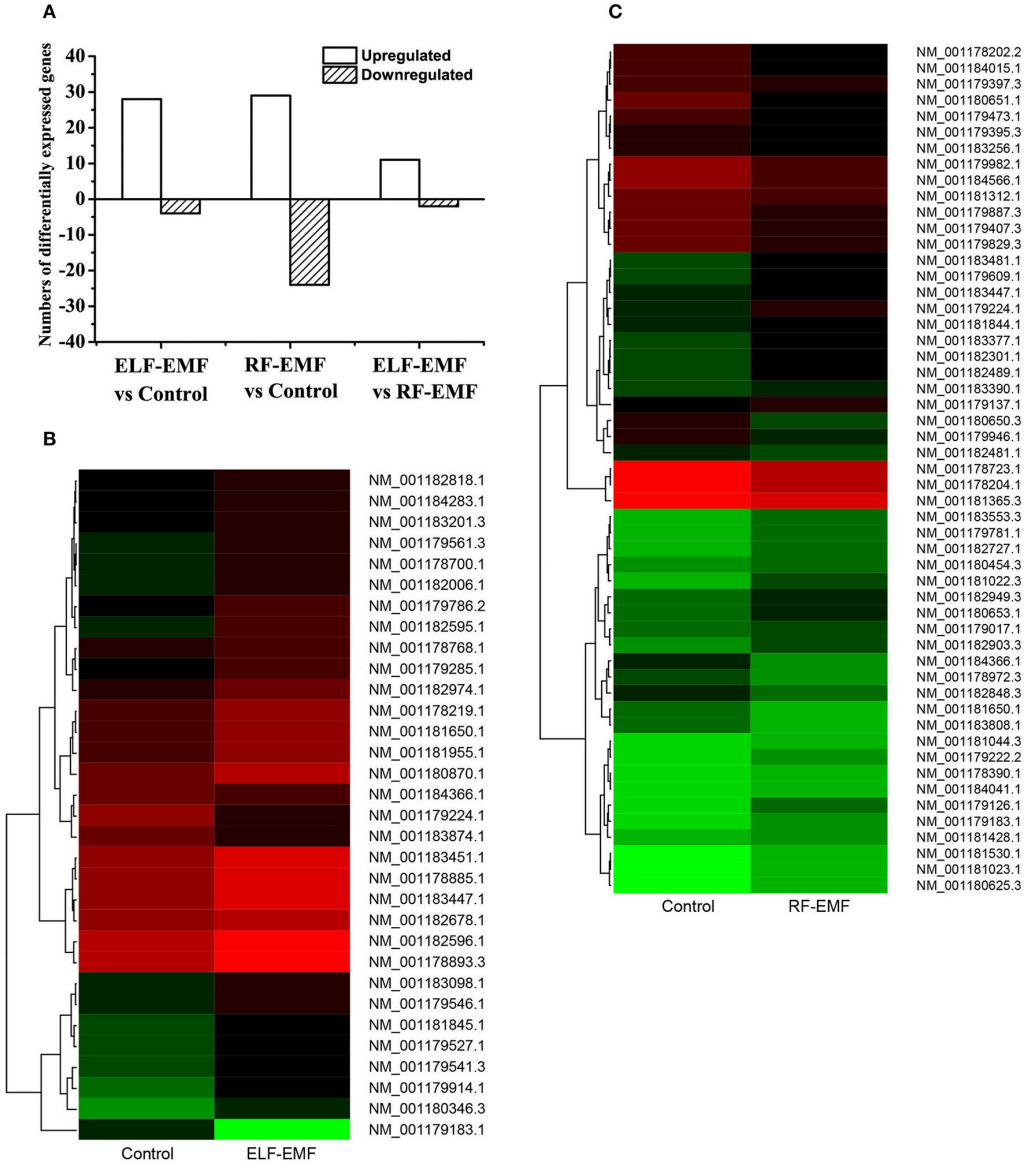
**Analysis of DEGs**. **(A)** Summary of the numbers of DEGs between the ELF-EMF exposure group, RF-EMF exposure group, and control group. **(B)** Cluster analysis of DEGs between the ELF-EMF exposure group and the control group. Each column represents an experimental condition, and each row represents a gene. Expression differences are shown in different colors. Red indicates upregulation, and green indicates downregulation. **(C)** Cluster analysis of DEGs between the RF-EMF exposure group and the control group. Each column represents an experimental condition, and each row represents a gene. Expression differences are shown in different colors. Red indicates upregulation, and green indicates downregulation.

To identify genes presenting significant changes in expression, differentially expressed tags were analyzed. The gene expression profile data analysis indicated that 28 genes were upregulated and four genes were downregulated under ELF-EMF exposure, whereas 29 genes were upregulated and 24 genes were downregulated under RF-EMF exposure (differences in expression levels >2.0-fold and < 0.5-fold, respectively; Figure 1A; see also Tables 1, 2). The detected changes in gene expression ranged from −5.86 to 2.95 fold (log_2_ ratio). A cluster analysis of gene expression patterns was performed (Figures 1B,C) to assess clustering models under the different experimental conditions, and the differences in reads per kilobase of transcript per million mapped reads (RPKM) between multiple different sample combinations were assessed.

**Table 1:** **DEGs in yeast cells under ELF-EMF exposure as confirmed by transcriptome analysis and RT-qPCR**.

**Gene name**	**Relative expression**			**DGE results**				
	**Control**	**ELF-EMF**	**Sig**.	**Control**	**ELF-EMF**	**Fold change**	***p*-value**	***q*-value**
***SNZ1***	**1** ± **0.04**	**2.33** ± **0.98**	^*^	**465.672**	**1568.87**	3.3690	**1.33E-135**	**3.33E-132**
*NOP16*	1 ± 0.14	1.38 ± 1.71	–	450.991	1179.19	2.6147	8.12E-73	4.51E-70
***CIN5***	**1** ± **0.19**	**2.87** ± **1.19**	^*^	**224.616**	**702.745**	3.1287	**1.42E-56**	**5.08E-54**
***HMS1***	**1** ± **0.17**	**2.03** ± **0.71**	^*^	**294.631**	**798.326**	2.7096	**1.17E-52**	**3.67E-50**
*DSF1*	1 ± 0.30	1.89 ± 2.25	–	277.486	729.555	2.6292	4.03E-46	1.06E-43
***FYV7***	**1** ± **0.17**	**2.23** ± **0.76**	^*^	**92.3254**	**329.906**	3.5733	**7.51E-32**	**1.50E-29**
***COG7***	**1** ± **0.08**	**2.35** ± **0.76**	^*^	**148.058**	**409.749**	2.7675	**1.04E-28**	**1.86E-26**
*DDR48*	1 ± 0.24	0.68 ± 0.03	^*^	244.842	547.054	2.2343	1.63E-26	2.55E-24
*HXT1*	1 ± 0.20	1.87 ± 0.62	^*^	226.27	68.9438	0.3047	1.70E-21	2.13E-19
*OLI1*	–	–	–	210.935	91.8825	0.4356	1.54E-12	9.42E-11
*REE1*	1 ± 0.42	0.77 ± 0.81	–	104.046	235.159	2.2601	1.58E-12	9.51E-11
*PAU7*	1 ± 0.20	0.79 ± 0.25		112.563	231.687	2.0583	2.29E-10	1.03E-08
***MCH2***	**1** ± **0.19**	**5.46** ± **4.32**	^*^	**32.6156**	**105.184**	3.2250	**4.15E-10**	**1.80E-08**
*EAF7*	1 ± 0.86	0.41 ± 1.03	–	63.4006	148.221	2.3378	6.67E-09	2.55E-07
***SNO1***	**1** ± **0.15**	**2.14** ± **0.84**	^*^	**29.9585**	**91.277**	3.0468	**1.98E-08**	**7.04E-07**
***MFM1***	**1** ± **0.15**	**0.58** ± **0.14**	^*^	**139.868**	**63.7499**	0.4558	**3.55E-08**	**1.22E-06**
*RTT107*	1 ± 0.23	0.55 ± 0.03	^**^	46.978	110.603	2.3544	4.59E-07	1.33E-05
*CUP1-2*	1 ± 0.08	3.45 ± 1.17	^**^	20.7793	0	0.0172	1.92E-06	4.85E-05
*DAK2*	1 ± 0.21	2.20 ± 1.37	–	7.267	36.5272	5.0264	5.06E-06	0.00012
*ENA5*	1 ± 0.04	2.34 ± 1.32	–	3.5604	27.7029	7.7808	5.74E-06	0.000132
*MPP6*	1 ± 0.25	0.55 ± 0.10	^**^	32.9707	79.9376	2.4245	1.06E-05	0.000228
*SRN2*	1 ± 0.28	0.53 ± 0.06	^*^	26.5533	69.2302	2.6072	1.29E-05	0.000273
*CTR86*	1 ± 0.15	0.59 ± 0.02	^**^	52.9865	106.685	2.0134	2.78E-05	0.00055
*PZF1*	1 ± 0.13	0.58 ± 0.10	^**^	30.8169	69.7851	2.2645	0.000115	0.001888
*YPS6*	1 ± 0.30	0.86 ± 0.28	–	29.6765	66.783	2.2504	0.000177	0.002677
*PEX34*	1 ± 0.09	0.66 ± 0.09	^*^	29.5107	65.312	2.2132	0.000267	0.003904
*GAS1*	1 ± 0.27	0.65 ± 0.36	–	32.9885	68.7931	2.0854	0.000453	0.006175
*LTO1*	1 ± 0.25	0.57 ± 0.13	^*^	23.8149	54.0532	2.2697	0.000668	0.008725
*PAU17*	1 ± 0.13	0.75 ± 0.42		17.8524	44.5345	2.4946	0.000749	0.00944
*INA22*	1 ± 0.27	0.63 ± 0.10	–	22.4756	51.0004	2.2691	0.000952	0.011432
***FLO11***	**1** ± **0.20**	**2.03** ± **0.47**	^*^	**11.6542**	**32.6193**	2.7989	**0.001564**	**0.016793**
*IST3*	1 ± 0.13	0.67 ± 0.12	^*^	15.1313	36.6169	2.4199	0.002922	0.027858

Black font indicates a concordant direction of change between DGE and RT-qPCR. ^“*”^ and ^“**”^ indicate P < 0.05 and P < 0.01 compared with the control, respectively. The error after “±” represents S.D.

**Table 2:** **DEGs in yeast cells under RF-EMF exposure as confirmed by transcriptome analysis and RT-qPCR**.

**Gene name**	**Relative expression**			**DGE results**				
	**Control**	**RF-EMF**	**Sig**.	**Control**	**RF-EMF**	**Fold change**	***p*-value**	***q*-value**
*SPG1*	1 ± 0.27	1.00 ± 0.34	–	16642	7502.02	0.4508	0	0
*ADY2*	1 ± 0.27	1.00 ± 0.46		10905.3	4732.48	0.4340	0	0
***GDH3***	**1** ± **0.13**	**0.57** ± **0.18**	^*^	**10497**	**4670.05**	**0.4449**	**0**	**0**
*HXT6*	1 ± 0.11	2.06 ± 0.67	^*^	1558.26	427.725	0.2745	8.89E-143	8.90E-140
*RRT15*	1 ± 0.47	1.05 ± 0.28		2483.52	1084.33	0.4366	7.55E-115	6.30E-112
*GAP1*	1 ± 0.06	2.62 ± 0.95	^*^	1581.78	598.356	0.3783	3.73E-95	1.87E-92
*IGD1*	1 ± 0.35	0.94 ± 0.34		2504.57	1217.77	0.4862	9.25E-92	3.86E-89
*HXT7*	1 ± 0.11	2.06 ± 0.67	^*^	777.278	198.276	0.2551	9.58E-78	3.00E-75
*RGI2*	1 ± 0.20	0.86 ± 0.35		1627.13	705.656	0.4337	7.27E-77	2.14E-74
*QCR9*	1 ± 0.06	1.71 ± 0.74	–	2048.54	1010.36	0.4932	7.48E-73	2.08E-70
*PCK1*	1 ± 0.19	1.09 ± 0.50	–	1686.78	785.697	0.4658	1.02E-68	2.43E-66
***HXT1***	**1** ± **0.20**	**1.98** ± **0.75**	^*^	**226.27**	**730.034**	**3.2264**	**3.02E-66**	**6.57E-64**
*YIG1*	1 ± 0.36	1.33 ± 0.90	–	1159.75	508.727	0.4387	4.41E-54	8.18E-52
*BDH1*	1 ± 0.42	0.90 ± 0.01	–	1166.92	524.102	0.4491	6.78E-52	1.21E-49
*IZH2*	1 ± 0.25	0.79 ± 0.09	–	892.157	383.719	0.4301	1.27E-43	1.88E-41
*ERG11*	1 ± 0.29	0.95 ± 0.15	–	420.558	893.478	2.1245	2.59E-43	3.71E-41
*SYG1*	1 ± 0.07	1.11 ± 0.23	–	1165.75	579.421	0.4970	1.96E-41	2.40E-39
*KGD1*	1 ± 0.03	1.99 ± 0.49	^*^	939.402	439.138	0.4675	9.67E-39	1.10E-36
***HXT3***	**1** ± **0.05**	**2.08** ± **0.99**	^*^	**81.897**	**331.14**	**4.0434**	**1.38E-38**	**1.53E-36**
***PIG2***	**1** ± **0.38**	**0.51** ± **0.03**	^*^	**893.721**	**433.105**	**0.4846**	**4.14E-34**	**3.83E-32**
*PHD1*	1 ± 0.12	1.59 ± 1.29	–	195.893	460.064	2.3485	1.38E-27	1.13E-25
*YOR29-13*	–	–	–	203.63	461.591	2.2668	4.57E-26	3.58E-24
*SSA2*	–	–	–	264.529	541.301	2.0463	5.49E-25	4.04E-23
*PAU5*	1 ± 0.27	0.87 ± 0.11	–	555.69	255.057	0.4590	1.68E-24	1.18E-22
*ERG13*	1 ± 0.12	1.02 ± 0.05	–	164.921	387.322	2.3485	1.76E-23	1.21E-21
***CYB5***	**1** ± **0.09**	**1.44** ± **0.09**	^**^	**113.507**	**299.672**	**2.6401**	**4.73E-22**	**2.96E-20**
*CIN5*	1 ± 0.19	2.11 ± 2.21	–	224.616	462.898	2.0608	6.59E-22	4.07E-20
*HRP1*	1 ± 0.26	0.99 ± 0.40	–	185.077	394.359	2.1308	3.82E-20	2.17E-18
*INA1*	1 ± 0.53	1.17 ± 0.34	–	176.524	367.757	2.0833	4.19E-18	2.14E-16
*OLI1*	–	–	–	210.935	69.378	0.3289	5.18E-17	2.49E-15
*PFK27*	1 ± 0.30	1.19 ± 0.19	–	156.473	315.607	2.0170	8.56E-15	3.63E-13
*ARI1*	1 ± 0.07	2.20 ± 1.95	–	41.3838	129.919	3.1394	9.64E-13	3.63E-11
***NGL3***	**1** ± **0.10**	**0.65** ± **0.16**	^*^	**319.488**	**157.792**	**0.4939**	**1.14E-12**	**4.20E-11**
*IDP3*	1 ± 0.13	2.11 ± 0.35	^**^	219.783	107.343	0.4884	2.23E-09	6.13E-08
***LCP5***	**1** ± **0.11**	**1.47** ± **0.17**	^*^	**83.5402**	**169.847**	**2.0331**	**9.37E-09**	**2.35E-07**
*SER3*	1 ± 0.28	1.64 ± 0.86	–	160.452	71.4007	0.4450	1.40E-08	3.46E-07
*PAU13*	1 ± 0.04	1.06 ± 0.03	–	26.0478	79.7762	3.0627	3.63E-08	8.57E-07
*AQR1*	1 ± 0.11	0.77 ± 0.05	^*^	76.5811	155.098	2.0253	4.67E-08	1.08E-06
*CUP1-2*	1 ± 0.08	1.72 ± 0.67	–	20.7793	65.5371	3.1540	3.74E-07	7.99E-06
*EKI1*	–	–	–	53.5796	115.698	2.1594	4.51E-07	9.53E-06
*REE1*	1 ± 0.42	1.06 ± 0.17	–	104.046	45.294	0.4353	2.98E-06	5.75E-05
*BAG7*	1 ± 0.09	1.21 ± 0.17	–	45.843	98.1491	2.1410	4.07E-06	7.68E-05
*URA1*	–	–	–	43.6743	92.7183	2.1229	8.98E-06	0.00015887
*ERG8*	1 ± 0.04	0.95 ± 0.23	–	41.4306	83.325	2.0112	7.08E-05	0.00101561
*FDH1*	1 ± 0.12	2.07 ± 1.89	–	94.3416	47.0511	0.4987	0.0001347	0.00179877
***ECM12***	**1** ± **0.09**	**1.55** ± **0.39**	^*^	**31.3301**	**62.8276**	**2.0053**	**0.0005835**	**0.00609889**
*HXT4*	1 ± 0.19	1.69 ± 0.76	–	21.4108	47.722	2.2289	0.0008326	0.00825492
*HIM1*	1 ± 0.15	2.22 ± 2.05	–	10.311	29.0254	2.8150	0.0016605	0.01440896
***TOS3***	**1** ± **0.10**	**1.19** ± **0.02**	^*^	**22.8827**	**46.9138**	**2.0502**	**0.0023268**	**0.01885189**
*PHS1*	1 ± 0.19	1.03 ± 0.27	–	12.136	30.1819	2.4870	0.0034477	0.02542343
*ALG5*	1 ± 0.19	1.20 ± 0.31	–	17.5805	37.5576	2.1363	0.0044534	0.03067166
***RCK1***	**1** ± **0.06**	**1.30** ± **0.11**	^*^	**12.0634**	**29.2301**	**2.4230**	**0.004844**	**0.03277581**
*CST26*	1 ± 0.17	1.03 ± 0.26	–	18.4392	37.7796	2.0489	0.0063213	0.03976224

Black font indicates a concordant direction of change between DGE and RT-qPCR. “^*^” and “^**^” indicate P < 0.05 and P < 0.01 compared with the control, respectively. The error after “±” represents S.D.

To determine whether ELF-EMF and RF-EMF exposure can affect patterns of gene expression, we analyzed genes within specific GO categories that were differentially expressed between the control and exposed yeast cells to characterize the potential *in vivo* responses of the yeast cells to ELF-EMF and RF-EMF. To enhance the GO enrichment analysis, clusters of DEGs (differences in expression levels of >2.0-fold or < 0.5-fold) were selected based on statistical criteria (*P* < 0.05).

**Figure 2:**
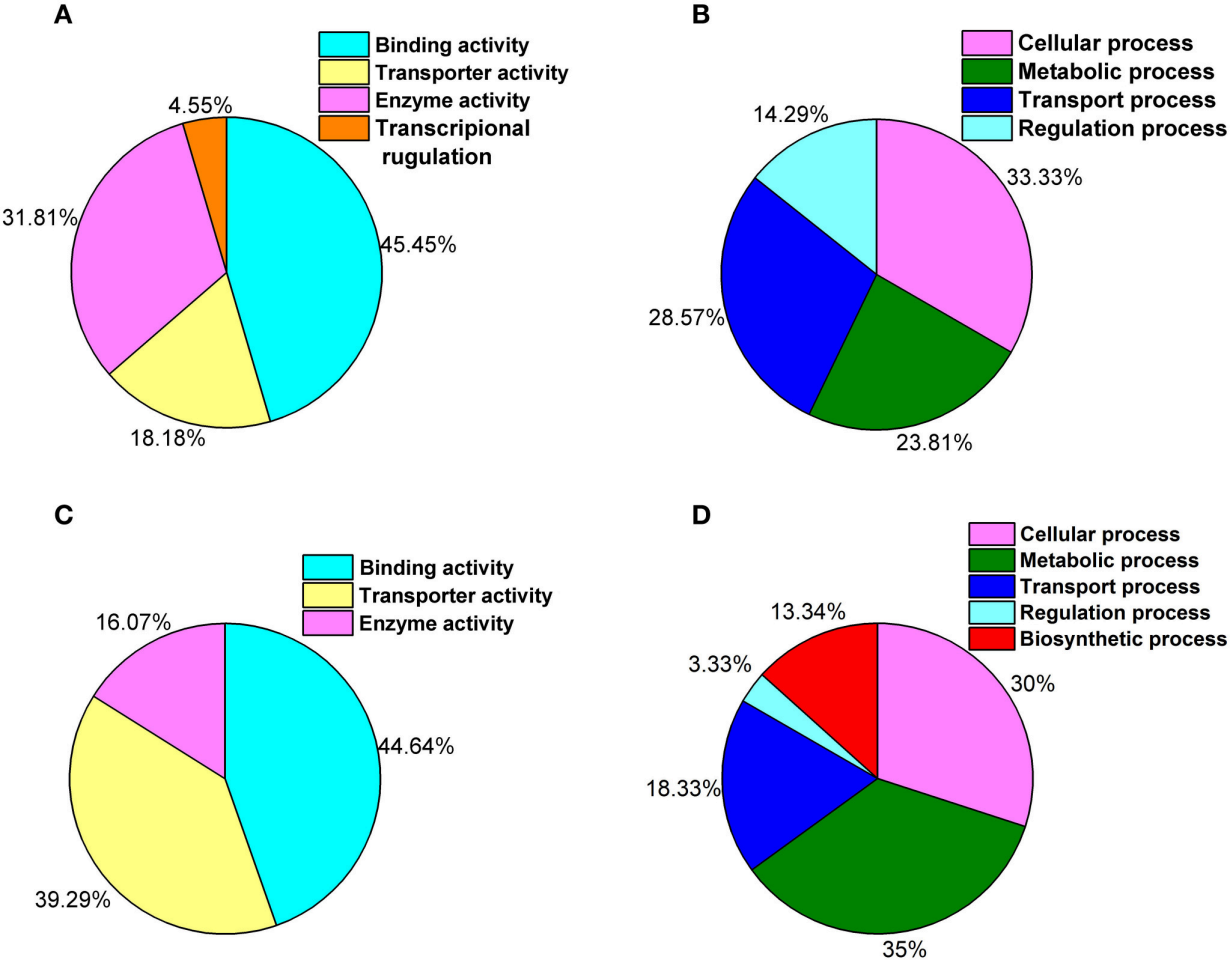
**GO classifications showing the putative functions of the DEGs**. **(A)** Percentage of genes involved in each molecular function under ELF-EMF exposure. **(B)** Percentage of genes involved in each biological process under ELF-EMF exposure. **(C)** Percentage of genes involved in each molecular function under RF-EMF exposure. **(D)** Percentage of genes involved in each biological process under RF-EMF exposure.

The comparison of both libraries and clustering by biological processes and molecular functions revealed that under ELF-EMF exposure, binding, and catalytic activity were among the most highly represented molecular function categories (Figure 2A), whereas the biological processes most commonly represented were cellular processes (Figure 2B). Interestingly, in the yeast cells exposed to ELF-EMF, only four genes, *HXT1, CUP1-2, MFM1*, and *OLI1*, which are relevant to ATP-binding and transmembrane transport, were downregulated significantly. Most of the DEGs detected under RF-EMF exposure were found to exhibit molecular functions of binding activity or transporter activity (Figure 2C) and are mostly involved in cellular processes and metabolic processes (Figure 2D).

### Confirmation of DEGs by RT-qPCR

To confirm the results of the DGE analyses, the expression levels of the DEGs were measured by RT-qPCR. Yeast cells were incubated, exposed and subjected to RNA isolation using the same process employed in DGE sample preparation. For the investigation of ELF-EMF exposure, a total of 31 genes were selected, and nine demonstrated a concordant direction of change in the DGE and RT-qPCR experiments (Table 1). Among the nine confirmed genes, the expression levels of eight, namely *SNZ1, CIN5, HMS1, FYV7, COG7, MCH2, SNO1*, and *FLO11*, were upregulated, whereas the expression level of only one gene, *MFM1*, was downregulated. For the analysis of RF-EMF exposure, a total of 48 genes were selected, and 10 genes demonstrated a concordant direction of change in the DGE and RT-qPCR results (Table 2). Among the 10 confirmed genes, the expression levels of seven (*HXT1, HXT3, CYB5, LCP5, ECM12, TOS3*, and *RCK1*) were upregulated, whereas the expression levels of three genes (*GDH3, PIG2*, and *NGL3*) were downregulated.

### Upregulation of glucose transport by ELF-EMF

**Figure 3:**
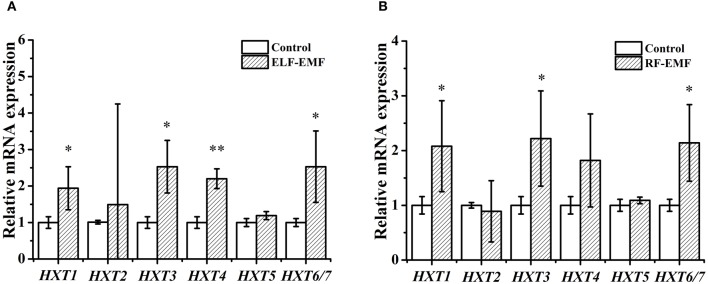
**Effects of ELF-EMF (A) and RF-EMF (B) exposure on the expression levels of genes involved in glucose transport**. All of the data represent the results from three independent experiments. Bars represent the S. D. of the mean. ^“*”^ and ^“**”^ indicate *P* < 0.05 and *P* < 0.01 compared with the control, respectively.

Cells need to consume energy to respond to external stress. The DGE analysis results (Tables 1, 2) revealed some genes encoding glucose transporters were significantly affected by ELF-EMF (*HXT1*) and RF-EMF (*HXT1, HXT3, HXT4, HXT6/7*). Yeast have at least six glucose transporters (Hxt1-5, and Hxt6/7) with various affinities for glucose (Kim et al., 2013). Therefore, we questioned whether the expression levels of other *HXT* genes were also changed. Six genes were investigated by RT-qPCR, and four genes were found to present significant changes compared with the control group (Figure 3A). Interestingly, high-affinity glucose transporters (*HXT4* and *HXT6/7*), a low-affinity glucose transporter (*HXT1*) and an intermediate-affinity glucose transporter (*HXT3*) were all upregulated after exposure to ELF-EMF.

Five genes encoding glucose transporters were significantly affected by RF-EMF exposure (Figure 3B). The expression of *HXT1, HXT3*, and *HXT4* was increased in both the DGE and RT-qPCR; however the increases in the *HXT1* and *HXT3* levels, but not the *HXT4* level, reached significance. The expression of *HXT6* and *HXT7* was decreased in the DGE results but increased significantly in the RT-qPCR results. In all, six *HXT* genes were investigated by RT-qPCR, and three genes were demonstrated to exhibit significant changes compared with the control group (Figure 3B). High-affinity glucose transporters (*HXT6/7*), a low-affinity glucose transporter (*HXT1*), and an intermediate-affinity glucose transporter (*HXT3*) were upregulated after exposure to RF-EMF.

### Expression of genes involved in the tricarboxylic acid (TCA) cycle, but not the glycolytic pathway, was enhanced by EMF exposure

After uptake of an increased amount of glucose, a cell must utilize metabolic pathways to transform the transported glucose into ATP or other available forms of energy. The glycolytic pathway and TCA cycle are two of the most important metabolic pathways in living organisms, generating reducing factors that drive the production of energy (Lee et al., 2011). We analyzed the transcription levels of genes related to glucose transporters, the glycolytic pathway and TCA cycle through RT-qPCR under ELF-EMF (Table 3) and RF-EMF (Table 4) exposure conditions. The transcriptome analysis and RT-qPCR did not find any significant changes in the glycolytic pathway under ELF-EMF or RF-EMF exposure. However, the expression of some genes encoding key enzymes in the TCA cycle was found to be significantly changed, as demonstrated through RT-qPCR analysis. In particular, the expression levels of *MFH2, LSC1, LSC2, IDP3*, and *KGD1* were significantly enhanced under ELF-EMF exposure conditions (Table 3). Under RF-EMF exposure, the expression levels of *DLS1, IDP3*, and *KGD1* were significantly increased, whereas the expression levels of *SDH1* and *OSM1* were decreased (Table 4).

**Table 3:** **Expression level of genes related to glucose transporters, the glycolysis pathway and the TCA cycle in yeast cells under ELF-EMF exposure**.

**Gene name**	**Relative expression**			**DGE results (fold change)**				
	**Control**	**ELF-EMF**	**Sig**.	**Control**	**ELF-EMF**	**Fold change**	***p*-value**	***q*-value**
***HXT1***	**1** ± **0.20**	**1.87** ± **0.63**	^*^	226.27	68.9438	0.3047	1.70E-21	2.13E-19
***HXT4***	**1** ± **0.19**	**2.14** ± **0.25**	^**^	21.4108	22.8704	1.0682	0.868956	0.964329
***PDH1***	**1** ± **0.13**	**2.33** ± **0.74**	^*^	304.369	452.58	1.4869	2.19E-07	6.71E-06
*IME1*	1 ± 0.49	2.06 ± 1.10	–	44.928	85.5195	1.9035	0.000478	0.00647
*QCR9*	1 ± 0.06	2.04 ± 1.23	–	2048.54	1615.72	0.7887	1.87E-14	1.44E-12
***GAP1***	**1** ± **0.06**	**2.51** ± **0.99**	^*^	1581.78	819.392	0.5180	2.84E-58	1.19E-55
*NOP16*	1 ± 0.14	1.39 ± 1.71	–	450.991	1179.19	2.6147	8.12E-73	4.51E-70
***HMS1***	**1** ± **0.17**	**2.03** ± **0.71**	^*^	294.631	798.326	2.7096	1.17E-52	3.67E-50
***SNZ1***	**1** ± **0.04**	**2.33** ± **0.99**	^*^	465.672	1568.87	3.3690	1.33E-135	3.33E-132
*FDH1*	1 ± 0.12	2.22 ± 1.54	–	94.3416	124.953	1.3245	0.0511523	0.2366150
***IDP3***	**1** ± **0.13**	**3.12** ± **0.92**	^**^	219.783	135.126	0.6148	3.05E-06	7.51E-05
***KGD1***	**1** ± **0.03**	**2.25** ± **1.00**	^*^	939.402	743.691	0.7917	3.18E-07	9.53E-06
*MCH2*	1 ± 0.17	2.28 ± 1.13	–	32.6156	105.184	3.2250	4.15E-10	1.80E-08
*ARI1*	1 ± 0.07	2.08 ± 1.24	–	41.3838	26.6397	0.6437	0.0626809	0.2706801
*HIM1*	1 ± 0.15	3.03 ± 3.40	–	10.311	16.193	1.5705	0.2688314	0.6234945
***COG7***	**1** ± **0.08**	**2.34** ± **0.76**	^*^	148.058	409.749	2.7675	1.04E-28	1.86E-26
***HXT3***	**1** ± **0.05**	**2.46** ± **0.75**	^*^	81.897	54.0016	0.6594	0.0125668	0.0855741
***HXT6/7***	**1** ± **0.12**	**2.41** ± **0.89**	^*^	1558.26	1712.18	1.0988	0.0259604	0.1458274
***MND2***	**1** ± **0.30**	**2.23** ± **0.71**	^*^	19.2026	38.4067	2.0001	0.0126255	0.0857408
***SNO1***	**1** ± **0.15**	**2.14** ± **0.84**	^*^	29.9585	91.277	3.0468	1.98E-08	7.04E-07
***POR1***	**1** ± **0.05**	**2.32** ± **0.27**	^**^	2034.46	2489	1.2234	4.86E-10	0.541238
***CIN5***	**1** ± **0.19**	**2.87** ± **1.19**	^*^	224.616	702.745	3.1287	1.42E-56	5.08E-54
*DSF1*	1 ± 0.29	1.89 ± 2.25	–	277.486	729.555	2.6292	4.03E-46	1.06E-43
***FYV7***	**1** ± **0.17**	**2.23** ± **0.76**	^*^	92.3254	329.906	3.5733	7.51E-32	1.50E-29
*DAK2*	1 ± 0.21	2.20 ± 1.37	–	7.267	36.5272	5.0264	5.06E-06	0.000119564
*SER3*	1 ± 0.29	1.45 ± 0.63	–	160.452	138.64	0.8641	0.1605830	0.4755729
*PCK1*	1 ± 0.19	1.62 ± 1.61	–	1686.78	990.534	0.5872	2.77E-44	6.93E-42
*CIT1*	1 ± 0.13	2.03 ± 1.03	–	815.743	1197.09	1.4675	3.17E-16	2.88E-14
*ENA*	1 ± 0.04	2.35 ± 1.32	–	3.5604	27.7029	7.7808	5.74E-06	0.0001324
***CUP1-2***	**1** ± **0.08**	**3.46** ± **1.17**	^**^	20.7793	0	0.0172	1.92E-06	4.85E-05
***FLO11***	**1** ± **0.20**	**2.03** ± **0.47**	^**^	11.6542	32.6193	2.7989	0.0015635	0.0167929
*DLS1*	1 ± 0.03	1.10 ± 0.15	–	81.8893	58.2187	0.7109	0.0356643	0.1812185
*PGI1*	1 ± 0.13	0.89 ± 0.11	–	388.319	549.588	1.4153	4.86E-07	1.40E-05
*PYK2*	1 ± 0.01	1.14 ± 0.29	–	61.2123	74.3659	1.2149	0.3003657	0.6605142
*PFK2*	1 ± 0.08	1.01 ± 0.06	–	140.43	137.877	0.9818	0.7722487	0.9363141
*PGK1*	1 ± 0.05	1.01 ± 0.38	–	366.105	537.763	1.4689	4.14E-08	1.41E-06
*MDH3*	1 ± 0.14	1.16 ± 0.15	–	451.42	597.273	1.3231	2.15E-05	0.000436713
*OSM1*	1 ± 0.09	1.12 ± 0.12	–	72.1792	72.8976	1.0100	0.9690338	0.9918229
***LSC1***	**1** ± **0.13**	**1.39** ± **0.09**	^*^	107.848	110.691	1.0264	0.9430152	107.848
*FRD1*	1 ± 0.01	1.08 ± 0.07	–	46.8417	55.3757	1.1822	0.4459918	0.7898758
*FBA1*	1 ± 0.01	0.92 ± 0.10	–	891.15	1304.04	1.4633	2.58E-17	2.63E-15
*PGD1*	1 ± 0.01	1.05 ± 0.18	–	54.7974	58.3441	1.0647	0.8052607	0.9440923
***GPD1***	**1** ± **0.03**	**1.25** ± **0.15**	^*^	4191.96	3732.62	0.8904	3.60E-09	1.42E-07
*ENO2*	1 ± 0.03	0.94 ± 0.51	–	223.019	314.854	1.4118	0.0001556	0.0024043
*SDH2*	1 ± 0.03	0.91 ± 0.32	–	2453.51	2395.62	0.9764	0.1607724	0.4755708
*LDH3*	1 ± 0.01	1.16 ± 0.24	–	97.5294	107.236	1.0995	0.5743998	0.8535840
*PDC6*	1 ± 0.03	0.97 ± 0.26	–	28.2676	17.5384	0.6204	0.0995088	0.3603776
*HXT5*	1 ± 0.10	1.19 ± 0.11	–	2786.44	2505.65	0.8992	8.13E-06	0.00017935
*PGM1*	1 ± 0.03	0.99 ± 0.06	–	40.8139	53.951	1.3219	0.20318046	0.542645776
*GLK1*	1 ± 0.09	1.07 ± 0.38	–	2020.46	1842.37	0.9119	0.0007317	0.009295
*MDH1*	1 ± 0.15	1.19 ± 0.06	–	638.802	808.16	1.2651	3.34E-05	638.802
*ACO1*	1 ± 0.01	0.70 ± 0.67	–	1149.07	1254.58	1.0918	0.0795965	0.3149256
*SDH1*	1 ± 0.17	1.21 ± 0.07	–	516.605	547.405	1.0596	0.4978283	0.8206952
*HXT2*	1 ± 0.05	2.43 ± 2.76	–	29.3301	41.7791	1.4244	0.158026	0.471346
*GPD2*	1 ± 0.18	1.16 ± 0.04	–	187.332	156.557	0.8357	0.069964	0.288917
*ADH7*	1 ± 0.27	1.10 ± 0.17	–	10.6131	19.4828	1.8357	0.11297	0.386211
*ADH1*	1 ± 0.14	0.94 ± 0.09	–	1448.17	1549.77	1.0702	0.158688	0.471355
*LDH1*	1 ± 0.05	1.08 ± 0.25	–	97.5294	107.236	1.0995	0.5744	0.853584
***LSC2***	**1** ± **0.03**	**1.52** ± **0.22**	^*^	169.371	188.673	1.1140	0.386568	0.749332
***MDH2***	**1** ± **0.03**	**1.39** ± **0.20**	^*^	315.628	352.627	1.1172	0.222194	0.568259

The expression levels were determined by both RT-qPCR and DGE. Black font indicates a significant change based on RT-qPCR results. ^“*”^ and ^“**”^ indicate P < 0.05 and P < 0.01 compared with the control, respectively. The error after “±” represents S.D.

**Table 4:** **Expression level of genes related to glucose transporters, the glycolysis pathway and the TCA cycle in yeast cells under RF-EMF exposure**.

**Gene name**	**Relative expression**			**DGE results (fold change)**				
	**Control**	**RF-EMF**	**Sig**.	**Control**	**RF-EMF**	**Fold change**	***p*-value**	***q*-value**
***HXT1***	**1.00** ± **0.16**	**2.08** ± **0.83**	^*^	226.27	730.034	3.2264	3.02E-66	6.57E-64
*HXT4*	1.00 ± 0.16	1.82 ± 0.85	–	21.4108	47.722	2.2289	0.0008325	0.0082549
*PDH1*	1.00 ± 0.13	1.85 ± 1.04	–	304.369	170.194	0.5592	5.34E-09	1.39E-07
*IME1*	1.00 ± 0.15	1.37 ± 0.33	–	44.928	29.9961	0.6676	0.1132011	0.2949002
*QCR9*	1.00 ± 0.06	1.83 ± 0.81	–	2048.54	1010.36	0.4932	7.48E-73	2.08E-70
***GAP1***	**1.00** ± **0.06**	**2.74** ± **1.94**	^*^	1581.78	598.356	0.3783	3.73E-95	1.87E-92
*NOP16*	1.00 ± 0.14	1.45 ± 1.15	–	450.991	640.025	1.4192	2.93E-10	8.96E-09
*HMS1*	1.00 ± 0.15	2.00 ± 1.49	–	294.631	408.725	1.3872	1.89E-06	3.80E-05
*SNZ1*	1.00 ± 0.15	2.23 ± 1.71	–	465.672	778.039	1.6708	2.02E-21	1.20E-19
*FDH1*	1.00 ± 0.11	2.61 ± 2.22	–	94.3416	47.0511	0.4987	0.0001347	0.0017987
***IDP3***	**1.00** ± **0.10**	**2.14** ± **0.36**	^**^	219.783	107.343	0.4884	2.23E-09	6.13E-08
***KGD1***	**1.00** ± **0.05**	**2.03** ± **0.53**	^*^	939.402	439.138	0.4675	9.67E-39	1.10E-36
*MCH2*	1.00 ± 0.21	2.56 ± 1.88	–	32.6156	35.856	1.0994	0.5936061	0.7840110
*ARI1*	1.00 ± 0.05	2.64 ± 2.38	–	41.3838	129.919	3.1394	9.64E-13	3.63E-11
*HIM1*	1.00 ± 0.12	2.79 ± 2.33	–	10.311	29.0254	2.8150	0.0016604	0.0144089
*COG7*	1.00 ± 0.08	2.61 ± 1.57	–	148.058	165.375	1.1170	0.1998005	0.4235399
***HXT3***	**1.00** ± **0.16**	**2.22** ± **0.87**	^*^	81.897	331.14	4.0434	1.38E-38	1.53E-36
***HXT6/7***	**1.00** ± **0.11**	**2.14** ± **0.70**	^*^	1558.26	427.725	0.2745	8.89E-143	8.90E-140
*MND2*	1.00 ± 0.25	2.09 ± 1.03	–	19.2026	15.3716	0.8005	0.5811724	0.7763955
*SNO1*	1.00 ± 0.23	1.86 ± 0.86	–	29.9585	39.51	1.3188	0.1969675	0.4200241
*POR1*	1.00 ± 0.05	1.73 ± 0.81	–	2034.46	1205.96	0.5928	1.20E-42	1.62E-40
*CIN5*	1.00 ± 0.20	2.66 ± 1.91	–	224.616	462.898	2.0608	6.59E-22	4.07E-20
*DSF1*	1.00 ± 0.30	2.58 ± 2.55	–	277.486	488.42	1.7602	3.99E-16	1.82E-14
***FYV7***	**1.00** ± **0.18**	**2.06** ± **0.79**	^*^	92.3254	106.658	1.1552	0.2082586	0.4353867
***DAK2***	**1.00** ± **0.27**	**2.09** ± **0.69**	^*^	7.267	12.2989	1.6924	0.2229699	0.4525376
*SER3*	1.00 ± 0.21	1.79 ± 0.96	–	48.6546	73.2877	1.5063	0.0152604	0.0755776
*PCK1*	1.00 ± 0.16	1.18 ± 0.56	–	1686.78	785.697	0.4658	1.02E-68	2.43E-66
*CIT1*	1.00 ± 0.10	1.77 ± 0.7	–	815.743	565.364	0.6931	8.99E-10	2.60E-08
*ENA*	1.00 ± 0.04	2.32 ± 1.58	–	454.088	391.099	0.8613	0.0949062	0.2610965
*CUP1-2*	1.00 ± 0.06	1.81 ± 0.74	–	20.7793	65.5371	3.1540	3.74E-07	7.99E-06
***FLO11***	**1.00** ± **0.18**	**2.30** ± **0.89**	^*^	11.6542	10.9914	0.9431	0.9540156	0.9842893
***DLS1***	**1.00** ± **0.03**	**1.17** ± **0.03**	^**^	42.3853	59.8834	1.4128	0.0565246	0.1879275
*PGI1*	1.01 ± 0.13	0.97 ± 0.12	–	388.319	298.165	0.7678	0.0027081	0.0209899
*PYK2*	1.00 ± 0.02	1.15 ± 0.37	–	61.2123	64.4407	1.0527	0.6309652	0.8081972
*PFK2*	1.00 ± 0.08	0.96 ± 0.10	–	140.43	133.875	0.9533	0.9110662	0.9631986
*PGK1*	1.00 ± 0.05	1.04 ± 0.12	–	366.105	350.739	0.9580	0.9087224	0.9623462
*MDH3*	1.00 ± 0.02	1.10 ± 0.29	–	451.42	473.676	1.0493	0.2098539	0.4370792
***OSM1***	**1.00** ± **0.10**	**0.71** ± **0.11**	^*^	72.1792	62.946	0.8721	0.5515330	0.7545153
*LSC1*	1.01 ± 0.13	1.12 ± 0.28	–	107.848	154.757	1.4350	0.0014792	0.0132020
*FRD1*	1.00 ± 0.01	1.01 ± 0.05	–	46.8417	41.6021	0.8881	0.6920109	0.8473706
*FBA1*	1.00 ± 0.01	0.87 ± 0.20	–	891.15	915.045	1.0268	0.1963018	0.4205748
*PGD1*	1.00 ± 0.01	1.10 ± 0.54	–	54.7974	102.197	1.8650	5.75E-05	54.7974
*GPD1*	1.00 ± 0.03	1.21 ± 0.37	–	4191.96	4608.05	1.0993	1.36E-09	3.82E-08
*ENO2*	1.00 ± 0.04	0.92 ± 0.31	–	223.019	232.394	1.0420	0.4204765	0.6511988
***SDH2***	**1.00** ± **0.03**	**0.76** ± **0.10**	^*^	2453.51	1395.84	0.5689	1.54E-58	2.96E-56
*LDH3*	1.00 ± 0.02	0.98 ± 0.10	–	97.5294	118.489	1.2149	0.0931550	0.2585518
*PDC6*	1.00 ± 0.03	0.94 ± 0.19	–	285.572	298.673	1.0459	0.3386051	0.5806149
*HXT5*	1.00 ± 0.11	1.09 ± 0.06	–	2786.44	2285.65	0.8203	5.63E-09	1.46E-07
*PGM1*	1.00 ± 0.04	0.99 ± 0.01	–	40.8139	57.6514	1.4125	0.0614598	0.1989200
*GLK1*	1.00 ± 0.09	0.96 ± 0.31	–	2020.46	1504.87	0.7448	1.42E-14	5.94E-13
*MDH1*	1.00 ± 0.15	0.96 ± 0.20	–	638.802	527.099	0.8251	0.0071345	0.0432999
*ACO1*	1.00 ± 0.02	0.88 ± 0.04	–	1149.07	696.726	0.6063	6.07E-23	3.94E-21
*SDH1*	1.00 ± 0.17	0.91 ± 0.16	–	516.605	410.33	0.7943	0.0029313	0.0224769
*HXT2*	1.01 ± 0.05	0.89 ± 0.56	–	29.3301	43.459	1.4817	0.070846	0.218427
*GPD2*	1.02 ± 0.18	1.05 ± 0.12	–	187.332	207.802	1.1093	0.170346	0.386987
*ADH7*	1.00 ± 0.26	1.08 ± 0.11	–	10.6131	13.5607	1.2777	0.493752	0.715134
*ADH1*	1.00 ± 0.15	0.81 ± 0.31	–	1448.17	1702.02	1.1753	3.97E-08	9.30E-07
*LDH1*	1.00 ± 0.05	1.06 ± 0.02	–	97.5294	118.489	1.2149	0.093155	0.258552
*LSC2*	1.00 ± 0.04	1.21 ± 0.23	–	169.371	177.585	1.0485	0.446852	0.674319
*MDH2*	1.00 ± 0.03	1.16 ± 0.35	–	315.628	404.879	1.2828	0.000151	0.001987

The expression levels were determined by both RT-qPCR and DGE. Black font indicates a significant change based on RT-qPCR results. ^“*”^ and ^“**”^ indicate P < 0.05 and P < 0.01 compared with the control, respectively. The error after “±” represents S.D.

## Discussion

The Illumina sequencing technology could help in finding potential responding gene candidates. However, for a weak stimulus such as EMF, the changes detected by Illumina sequencing are usually very small, therefore, it is important and essential to further examine all statistically significantly affected genes with RT-qPCR to confirm all positive changes and to exclude all false positives. To validate the DGE data, we examined the expression of all the DEGs identified under ELF-EMF and RF-EMF exposure through RT-qPCR. The changes observed in the DGE analysis were generally larger than those obtained by RT-qPCR. This was also seen in previous analysis of gene expression profiles using DGE and RT-qPCR (Luan et al., 2011) and in analysis using microarray and RT-qPCR (Chen et al., 2012). The inconsistency may be caused by the lower sensitivity of RT-qPCR compared with DGE analysis. On the other hand, the greater variation in the DGE analysis may reflect the relative difficulty in obtaining biological replicates using Illumina sequencing technology compared with RT-qPCR. The data obtained by RT-qPCR with three replicates might show less variability than those obtained from Illumina sequencing technology with only one biological replicate. Therefore, where the results obtained through DGE and RT-qPCR were different, greater emphasis were given to the RT-qPCR results. The genes involved in glucose transport and the TCA cycle were analyzed mainly based on the RT-qPCR results.

Global gene expression in *Saccharomyces cerevisiae* had been analyzed previously to evaluate the effects of ELF-EMF and RF-EMF exposure (Chen et al., 2012). They exposed yeast cells for 6 h to either 0.4-mT 50-Hz ELF-MF or 1800-MHz RF-EMF at a SAR of 4.7 W/kg. Gene expression was analyzed by microarray screening and confirmed by RT-qPCR. After exposed to ELF-MF, only three genes were found to be upregulated by 1.3-fold in the GeneChip assay, and none were confirmed by RT-qPCR. After exposed to RF-EMF, 13 genes showed downregulation, and 27 genes showed upregulation in the GeneChip assay. However, only two genes, *SMC3* (YJL074C, 1.20 ± 0.17) and *AQY2 (m)* (YLL053C, 1.08 ± 0.25) were confirmed by RT-qPCR (*P* < 0.05). In our study, the expression of SMC3 and *AQY2 (m)* under RF-EMF were also checked by RT-qPCR, and the expression of *AQY2 (m)* (1.46 ± 1.02) and *SMC3* (0.62 ± 0.33) was not significantly altered, compared with control group (1.00 ± 0.23). The differences in exposure strength and exposure duration might be one of the reasons why we got different results. As a weak stimulus, EMF usually exhibits very weak biological effects, and is sensitive to cell type and physical conditions. Numerous results showed diverse gene expression profile in various organisms and under different exposure conditions, therefore, further studies on the biological effects of RF-EMF and ELF-EMF are required and equal attention should be paid to the negative reports as to the positive ones.

The transcriptome analysis revealed the upregulation of two genes involved in the vitamin B6 metabolism pathway, namely *SNO1* and *SNZ1*, under ELF-EMF exposure, and these genes showed a concordant direction of change by RT-qPCR (Table 1). Unlike mammals, most unicellular organisms and plants are prototrophic for vitamin B6. The vitamin B6 biosynthesis pathway depends on the products of the *SNZ1* (also referred to as *PDX1*) and *SNO1* (also referred to as *PDX2*) genes (Rodríguez-Navarro et al., 2002). *SNZ1*, a member of a highly conserved gene family, was first identified through studies of proteins synthesized in stationary-phase yeast cells (Braun et al., 1996). The highly conserved gene *SNO1* (*SNZ1* proximal Open reading frame) is located downstream of *SNZ1*, and its regulation is coordinated with the neighboring gene through the same promoter (Uppuluri et al., 2006). Snz proteins contain no distinct functional motifs but exhibit very distant relationships with proteins involved in amino acid, vitamin, and nucleic acid biosynthesis (Dong et al., 2004). Nevertheless, Snz-related proteins do appear to have a role in stress responses. For example, in the rubber tree *Hevea brasiliensis*, expression of an SNZ-related gene can be induced in response to ethylene and salicylic acid (Sivasubramaniam et al., 1995). In the fungus *Cercospora nicotianae*, the Snz1-related protein Sor1 is required for resistance to singlet oxygen-generating photosensitizers (Ehrenshaft et al., 1998). Sor1 is also required for resistance to photosensitizing toxins in the fungus *C. nicotianae* (Ehrenshaft et al., 1999). The upregulation of *SNO1* and *SNZ1* indicates that ELF-EMF may affect cellular oxygen stress, which is consistent with the results obtained in other studies. For example, Frahm et al. reported that 24 h of exposure to 50-Hz ELF-MF at 1 mT elevated phagocytic activity and the intracellular ROS level in the absence of any genotoxic effects (Frahm et al., 2010). In addition, 50-Hz ELF-EMF is able to potentiate the cellular damage induced by oxidative stress in humans (Tiwari et al., 2015).

Yeast have at least six glucose transporters (Hxt1-5, Hxt6/7) with various affinities for glucose (Kim et al., 2013). *HXT* genes are induced by glucose, but yeast expresses only the glucose transporters best suited to the amount of glucose available in the environment (Pasula et al., 2007). The low-affinity transporter gene *HXT1* is expressed only in the presence of high concentrations of glucose (Tomás-Cobos et al., 2005; Kim et al., 2013). In contrast, the expression of high-affinity transporter genes, such as *HXT2, HXT4, HXT5*, and *HXT6/7*, is induced by low levels of glucose (Kasahara et al., 2006; Kim et al., 2013). It is widely known that expression of the Hxt1 transporter in the presence of high levels of glucose leads to a reduction in the concentration of glucose in the culture medium due to glucose uptake by Hxt1, which in turn leads not only to induced expression of high-affinity glucose transporters but also to repression of Hxt1 (Roy et al., 2015). This regulatory property of glucose transporters hampers the development of effective methods to determine the differential expression of different *HXT* genes in response to different levels of glucose (Ozcan and Johnston, 1995; Pasula et al., 2007; Salema-Oom et al., 2011). Among the six *HXT* genes, the expression of four genes and three genes was enhanced under ELF-EMF exposure and RF-EMF exposure, respectively (Figure 3). These results suggest that ELF-EMF and RF-EMF exposure may improve the transport of glucose and consequently increase the intracellular glucose concentration, which might enhance the downstream glycolytic pathway and TCA cycle.

**Figure 4:**
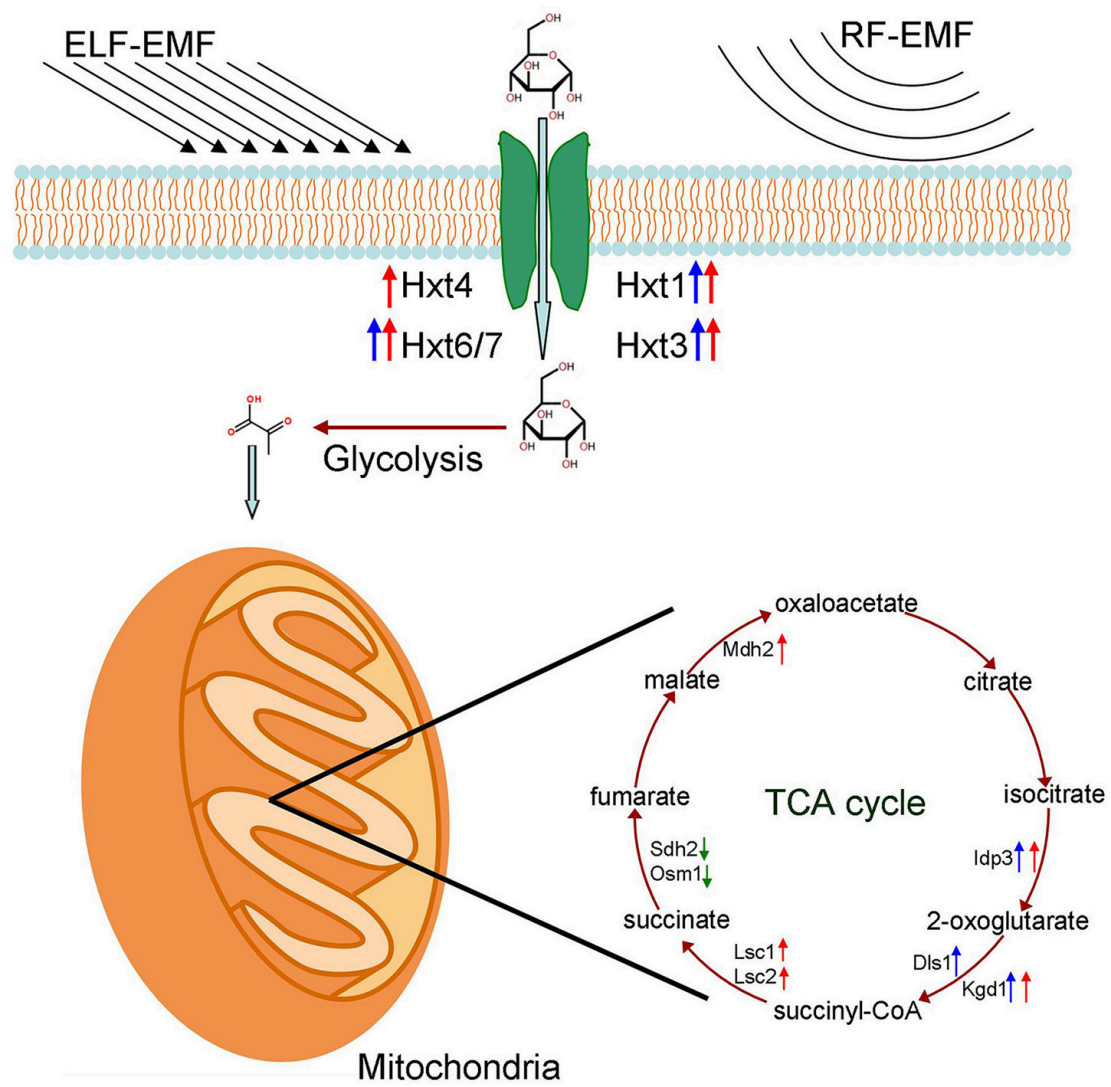
**Effects of ELF-EMF and RF-EMF exposure on the expression levels of genes involved in glucose transport and the TCA cycle**. Red arrows represent a significant increase in transcription under ELF-EMF exposure. Blue arrows represent a significant increase in transcription under RF-EMF exposure. Green arrows represent a significant decrease in transcription under RF-EMF exposure.

The transcription levels of genes related to glucose transport, the glycolysis pathway and TCA cycle under ELF-EMF and RF-EMF exposure were measured by RT-qPCR (Tables 3, 4). Although we did not find any significant changes in expression of genes involved in the glycolytic pathway, the expression levels of some genes encoding key enzymes in the TCA cycle were found to be significantly upregulated (Figure 4). The *IDP3* gene encodes peroxisomal NADP-dependent isocitrate dehydrogenase (ICDH), which catalyzes the oxidation of isocitrate to form alpha-ketoglutarate and NAD(P)H (Lu and McAlister-Henn, 2010). Environmental stresses, such as nutrient stress, temperature alteration, and tobacco smoking, have been shown to modulate ICDH activity (Konga et al., 2009; Jaafar et al., 2015). NAD(P)H is an effective scavenger of free radicals and hydrogen peroxide; thus ICDH might also act as an indirect antioxidant by providing NAD(P)H, which is required in corneal defense against oxidative damage (Lassen et al., 2008). Another key gene is *KGD1* encoding a subunit of the mitochondrial alpha-ketoglutarate dehydrogenase complex, which catalyzes the oxidative decarboxylation of alpha-ketoglutarate to form succinyl-CoA (Samokhvalov et al., 2004). *KGD1* has been shown to exhibit increased activity *in vitro* upon the addition of Aluminium lactate (Hamel and Appanna, 2001). However, a marked reduction in *KGD1* activity has been observed in neurological diseases and has been postulated to occur due to free radical formation and abnormalities in metal metabolism (Gibson et al., 2000).

Interestingly, while the expression of genes involved in glucose transport and TCA cycle were upregulated under exposure, the expression of genes involved in glycolysis were not found to be elevated significantly. One reason might be that the glycolytic pathway under this condition was adjusted mainly via the allosteric regulation and covalent modification regulation on expressed enzymes, rather than the regulation on gene expression levels. Therefore, the expression level of genes involved in glycolysis did not exhibit significant alteration. In addition, several homology genes are predicted to be involved in the 10 enzymatic reactions of glycolytic pathway. Thus, the redundancy in homology genes might make some contribution to the lack of detectable alteration in gene expression level. Considering the two reasons above and the relative weak effect of EMF exposure, it was still possible that glycolysis was enhanced to some extent under our exposure condition, even though significant alteration in expression levels of genes for glycolysis were not detected by RT-qPCR in this study.

In addition to propelling the production of energy, the TCA cycle also produces reservoirs of essential metabolic precursors that are channeled toward the biogenesis of essential compounds such as amino acids, fatty acids and carbohydrates (Lee et al., 2011). This cycle can be modified, and enzymes can be upregulated or downregulated depending on the needs of the cell. The demand for energy necessitates the complete decarboxylation of acetyl CoA and the formation of reducing moieties such as FADH_2_ and NADH (Rezaei et al., 2015). Environmental stress, whether physical and/or chemical in nature, can have a major impact on this metabolic cycle (Rezaei et al., 2015). Enzymes involved in the TCA cycle that are overexpressed under ELF-EMF and RF-EMF exposure might accelerate TCA reactions and increase the production of energy and some key molecular components, and both of these outcomes will help the cells meet their requirements in response to environmental stress.

In this study, the expression levels of some key genes involved in glucose transport and the TCA cycle were found to be increased under ELF-EMF and RF-EMF exposure (summarized in Figure 4), which suggest the available energy in cells may be improved consequently. Metabolism was also found to be enhanced under EMF exposure in various model organisms in our previous work (Li et al., 2013; Shi et al., 2015; Cai et al, unpublished data). The enhancement of metabolism might be a universal response of various organisms to EMF exposure because it would provide energy to elevate cellular processes of protection against ELF-EMF and RF-EMF exposure, increase the production of NAD(P)H to counteract the oxidative damage caused by ELF-EMF and RF-EMF, and ultimately improve the resistance of cells to ELF-EMF and RF-EMF.

In recent decades, numerous studies have shown that ELF-EMF and RF-EMF exposure were associated with cancer diseases (Calvente et al., 2010). Most cancer cells show an enhanced nutrients importation and consumption, which could provide energy and biosynthetic intermediates for rapid proliferation of cancer cells. Our finding of enhanced expression of energy metabolism related genes in yeast cells under EMF exposure is consistent with the elevated importation and catabolism in cancer cells, and may provide some clues for research on the mechanism underlying carcinogenicity of EMF exposure.

In cancer cells, the glucose uptake and glycolysis were elevated, however, the TCA cycle was not enhanced. Instead of entering TCA cycles, many glycolytic intermediates leave glycolysis and take part in diverse biosynthetic reactions, such as pentose phosphate pathway, hexosamine biosysthesis, glycerol-3 phosphate biosynthesis, one-carbon cycle, and lactate biosynthesis (Pavlova and Thompson, 2016). The reprogramming of carbon metabolism in cancer cells reaches a new balance between energy metabolism and compounds biosynthesis. This new balance will better meet the requirement of high demand of biosynthetic intermediates for rapid proliferation, and low oxygen consumption in the hypoxia microenvironment of cancer cells (Pavlova and Thompson, 2016). In addition, this new balance has lower reactive oxygen species (ROS) production and higher NADPH production, and is beneficial to the resistance of oxidative stress (Sosa et al., 2013). In our study, the expression of proteins involved in glucose transport and TCA cycle were both enhanced in the yeast cells under EMF exposure. Compared with control cells, the exposed yeast cells were incubated in similar conditions except for exposure, and exhibited similar proliferation speed. Therefore, the yeast cells under EMF exposure, unlike cancer cells, did not require significantly more biosynthetic intermediates and lower oxygen consumption. However, the elevated TCA cycle in exposed yeast cells could produce more ROS, which may damage macromolecules and cell processes, and lead to various diseases, including cancer. Moreover, the elevated metabolism may increase the levels of a number of key metabolites including SAM, acetyl-CoA, NAD(+), and ATP, which could serve as essential co-factors for many, perhaps most, epigenetic enzymes that regulate DNA methylation, posttranslational histone modifications, and nucleosome position (Donohoe and Bultman, 2012). Thus, the elevated metabolism under EMF exposure may contribute to the carcinogenicity of EMF exposure.

It remains unclear whether EMF exerts a biological impact. The biological effects of EMF at its usual strength are generally weak and might be easily masked by the cellular response systems. However, the elevated metabolism observed in this study reveals that ELF-EMF and RF-EMF exposure do have some effects. The effect of EMF on cellular response systems, including metabolism, should be prioritized in future investigations.

## Author contributions

KL and HL performed all of the biological experiments. CY, HL, and KL constructed and maintained the exposure systems. HL conceived the study. HL, KL, and PC wrote the manuscript. PC led the project and reviewed the manuscript.

## Funding

This work was supported by the Scientific Equipment Development Project of Chinese Academy of Sciences (CAS) (YZ201104, YZ201205), the Innovation Funds of Institute of Urban Environment, CAS (09L6321A90), the Xiamen Science and Technology Plans Project (3502Z20126012), the National Natural Science Foundation of China (31270888) and the Natural Science Foundation of Fujian Province (2012J01157).

### Conflict of interest statement

The authors declare that the research was conducted in the absence of any commercial or financial relationships that could be construed as a potential conflict of interest.
