# Supplementary material for: Exposure of ELF-EMF and RF-EMF Increase the Rate of Glucose Transport and TCA Cycle in Budding Yeast
Source: Front Microbiol. 2016 Aug 31;7:1378. doi: 10.3389/fmicb.2016.01378 (PMC5005349; doi:10.3389/fmicb.2016.01378)
Supplement: Supplementary file 1 [file Table1.DOC]

Table.S1 Primers used in this study.

| Gene name | Primer sequence（5’-3’） |
| --- | --- |
| *LDH1-FOR* | TTGCTTTGCGTAAACAACT |
| *LDH1-REV* | ACTACACCAGGGACGGATT |
| *LDH2-FOR* | ATAGTTTGCCCTGGCGTTGT |
| *LDH2-REV* | TAACTCTTGGTCTGCCGATGG |
| *MDH1-FOR* | GGTCAAGGGGTTTACTCCAG |
| *MDH1-REV* | CGTGTCATACCAGGCTTTCT |
| *MDH2-FOR* | GTCGTTATCGCTGCTTTTGA |
| *MDH2-REV* | GTTGATGGCTTCTTGGTTGA |
| *MDH3-FOR* | GTTTGGTCCCTATTGCTGTG |
| *MDH3-REV* | TACCAGGTCAAGGTTCGTCA |
| *SDH1-FOR* | CCCATACGGTTGCTGACACT |
| *SDH1-REV* | TTGCGATGGATTCTTTACCC |
| *SDH2-FOR* | CAAAGGATGGAACGGAAGTG |
| *SDH2-REV* | ACGATGGACATGAAGTAGAGCAG |
| *LSC1-FOR* | AAAAGGCGGGTCAAACAC |
| *LSC1-REV* | GGAGGAACAAAGATGGCACT |
| *LSC2-FOR* | TCCTGGAGGGACTTATCACA |
| *LSC2-REV* | AAACCAGCACCATTGACTAA |
| *SNC1-FOR* | GAAGATAAAGCCGATAACCTA |
| *SNC1-REV* | TGATTACTAAAGCCAGACACA |
| *SNC2-FOR* | CAACAGGGTCAGAAAGCAAA |
| *SNC2-REV* | TGGACGACGATAGGAACGA |
| *DLS1-FOR* | TCGCCCAAGTTACCTGTAGA |
| *DLS2-REV* | GAATGCGTCATCCGAAGTG |
| *ACO1-FOR* | CATTGCGGGTGATTTGAGA |
| *ACO1-REV* | CAGGTGGAGCTTGGTAAGTGT |
| *FRD1-FOR* | CCGAGACTCAACGTCACTTC |
| *FRD1-REV* | TGGACACCTTTACCTTTAGCAG |
| *OSM1-FOR* | GTTTAGGCGGCATCTTATTG |
| *OSM1-REV* | TCTCGTAGACTTTGTCGCTCA |
| *HXT2-FOR* | TCGTAAGTGTCTATTGGGTGG |
| *HXT2-REV* | AGCCTTGGAAGATGGTTGA |
| *HXT5-FOR* | TGCTTTTGGTGGGTTCGTGT |
| *HXT5-REV* | GGTTCTGACATCGGAAAGATAGGT |
| *GLK1-FOR* | GGTCATCGGATGTATTTTCGG |
| *GLK1-REV* | ACTCCTGTGGCAACTTCGTG |
| *PGI1-FOR* | ACTAACGCTAACACTGCCA |
| *PGI1-REV* | GTAACGACCACCGACCC |
| *PFK2-FOR* | CTAAATGCTATGAACGCTCCTC |
| *PFK2-REV* | GTTACCTAATGGGTCCAAAGTG |
| *FBA1-FOR* | GTGAACCATTATTCTCCTCCCA |
| *FBA1-REV* | TACCGATTTCCATTTCTAACCA |
| *GPD1-FOR* | GGCATCACTCTACCCGACA |
| *GPD1-REV* | GCACCAACTTCAAAACCCT |
| *GPD2-FOR* | GGGATGGGGTAACAATGCC |
| *GPD2-REV* | CCAGCGGATTCTTGATAGTAGG |
| *PGK1-FOR* | ACTCTTCTATGGTCGGTTTCG |
| *PGK1-REV* | TGGTCTGGTTGGGTTCTCC |
| *PGM1-FOR* | TGTTGGAGGAGATGGTCG |
| *PGM1-REV* | ACTTTCTGACACCGTTTGC |
| *ENO2-FOR* | TGTTATGGTTTCCCACAGAT |
| *ENO2-REV* | TGGTTCAACTTAGCCAATCT |
| *PYK2-FOR* | CTGTATGAACCGAAACGCCT |
| *PYK2-REV* | TCCACCATTCCGAAAGACC |
| *PDC-FOR* | ACGGTCCAAAGGCTCAATA |
| *PDC-REV* | GCGACTCTGTGGGTTTCATA |
| *PDC6-FOR* | TTTAGGTGCTGCCTTTGCC |
| *PDC6-REV* | GTGGAGATTTCTTGGACGGTTA |
| *URE2-FOR* | ACGGATGAGGTTAGAAGAGTTT |
| *URE2-REV* | CTACAAGCCATACGGGATAAT |
| *ADH7-FOR* | GCCTTGGCGTGTTTTGAGT |
| *ADH7-REV* | TGTGAAATGTAGCCGTCCTT |
| *ADH1-FOR* | GACTTCTTCGCCAGAGGTTT |
| *ADH1-REV* | TCTACCAACGATTTGACCCT |
| *HXT1-FOR* | TTTGGTTGAAGCTGGCAGAA |
| *HXT1-REV* | TGTATGGATGGTCAGGTGGG |
| *HXT3-FOR* | ATGGGCTGTATGGTTTTCG |
| *HXT3-REV* | GCATCGTAGTTAGCACCTCTT |
| *HXT4-FOR* | CGCCTACGTTACAGTTTCC |
| *HXT4-REV* | CCATCGTGGTGCTTCATA |
| *HXT6/7-FOR* | TGAAGGTGAAGAGCACGAAC |
| *HXT6/7-REV* | GTATCCCAACCGAAAACGAA |
| *PHD1-FOR* | AGGTTAGGGAAGTCGTGAAG |
| *PHD1-REV* | GAAGAGCGGGTACAGATGAT |
| *MND2-FOR* | ATAGCGAATACAGCCAAGAGC |
| *MND2-REV* | CCCTAGTCCAATTCCTCCAA |
| *IME1-FOR* | CATACACTCCAGGATTATACGA |
| *IME1-REV* | TTGCTCCATACATTCCATTAC |
| *SNO1-FOR* | CTCATCGCTCAAAGAACAGG |
| *SNO1-REV* | CCAGCACAAGTACCCCAAA |
| *DDR48-FOR* | AGCAAAATCGGAGAGGACAGA |
| *DDR48-REV* | CGTTGTTGTTGGAGTCGTTGT |
| *REE1-FOR* | GTTTATGGCGGAAATGCTC |
| *REE1-REV* | TTGGTCCAACAAGGTAATGGT |
| *PAU7-FOR* | GTCAAATTAACTTCAATCGCTGCC |
| *PAU7-REV* | TAGTGGTTGCTGCGGCG |
| *EAF7-FOR* | AAGTTGAGCCAATCTTACA |
| *EAF7-REV* | TTGCTTGCGTACTCTTATT |
| *MFM1-FOR* | GGATAAGATACGCCATAGGAA |
| *MAM1-REV* | AGCCAAACAGTAGATATTAACCC |
| *RTT107-FOR* | ATAATAACAAAGAACTGGCTAC |
| *RTT107-REV* | TATTTCGATGTCAACCTCA |
| *MPP6-FOR* | TTTGGTAAGACGGATGACG |
| *MPP6-REV* | TATTGAGGTCCCACGCTGA |
| *SRN2-FOR* | CTTAACGGACTTTGCTGGA |
| *SRN2-REV* | TTCGGAATGGTAATCTTGC |
| *CTR86-FOR* | GAATAATGCCAAGAATCAGG |
| *CTR86-REV* | CTAATCTAACTTTCCCGCC |
| *PZF1-FOR* | TCTGTGACTATGATGGCTGTG |
| *PZF1-REV* | CTGAAATGCTCTTAAACCCTG |
| *YPS6-FOR* | ACACCCTGCCTATGCTGC |
| *YPS6-REV* | CTGGAAATTGAATATCCGAAAC |
| *PEX34-FOR* | GGGAAATGGTCTAGTAAAGGA |
| *PEX34-REV* | CTTGGAGTAAAGCAGCAGTAA |
| *GAS1-FOR* | CTGCCTCTTCTAACGCTTCGG |
| *GAS1-REV* | AGCGGCAATGGATAAGGAAAT |
| *LTO1-FOR* | ATTCCAAAGATTTACGCTCCT |
| *LTO1-REV* | CACACTTTCATCATCGTTATTCA |
| *PAU17-FOR* | ATGTTCCAAGCCGCTCATCC |
| *PAU17-REV* | AGCAGTGTAGATACCGTCTGC |
| *INA22-FOR* | TGGCAAAGTATAATAGACGAGG |
| *INA22-REV* | CAAATCAGTGACAATCCCTTC |
| *IST3-FOR* | TGGAGTCCCTGTAGATGTAAT |
| *IST3-REV* | TTGACCGTTGATCCTCATA |
| *SPG1-FOR* | TATTATGTTAGGGCTGGTGGG |
| *SPG1-REV* | GTTGTCTAAGCTATGTGCAGGAA |
| *ADY2-FOR* | CTGGTGGTGTCCTGGGAGTTG |
| *ADY2-REV* | GGTAATGGGAATGGACGAGCC |
| *GDH3-FOR* | CTACGCTTCCACCCATCAG |
| *GDH3-REV* | TCGTTGTCAGACTTGCCTTTC |
| *RRT15-FOR* | GGGGACTTTTACCCTTTTG |
| *RRT15-REV* | GGGCGGCACATCTGTTA |
| *IGD1-FOR* | GCAATACAGGACCACTAACGA |
| *IGD2-REV* | TGCCAGCACGAGAACAGAT |
| *RGI2-FOR* | CCGCTATCAACAAGCCAGAA |
| *RGI2-REV* | CGCCATAATTCCAAACAATCC |
| *YIG1-FOR* | CAAGTATGGCAACTACACGG |
| *YIG1-REV* | GCTAAAGGTAAGCTGGTCGA |
| *BDH1-FOR* | GCAGTTCAGCCTTGGTTCTT |
| *BDH2-REV* | ACCTCAACGCCCAGTTTCTT |
| *IZH2-FOR* | ATATTTGGGGTCATTCTCACC |
| *IZH2-REV* | GACAATCCCGTTCTCCATCT |
| *ERG11-FOR* | ACAAAGATTCTGCCTCCTC |
| *ERG11-REV* | ACCGAAAGGTAAGTATGGA |
| *SYG1-FOR* | AATGGCTGGTGGTTACTCTT |
| *SYG1-REV* | TGGCAATATCGGCAATAGA |
| *PIG2-FOR* | ATTAGGCGAGGAGGAAGAC |
| *PIG2-REV* | TGTAAATGGGAGGTGAAGG |
| *PAU5-FOR* | CTGGTATTCCCGCCGACCAA |
| *PAU5-REV* | AATAGTGTAGATACCGTCTGCGG |
| *ERG13-FOR* | TTGCTAAGCCATTCCACAA |
| *ERG13-REV* | AAAGGCGGCATAAACAGAT |
| *CYB5-FOR* | ACCTCTGAAAACCAAAGTAAAG |
| *CYB5-REV* | CAACAAATAATAAGCAACACCTA |
| *HRP1-FOR* | GGCACTGCGAATGACAAGG |
| *HRP1-REV* | TTGTTCCCAAGGTAAGCCAGA |
| *INA1-FOR* | ATTCCAACTTCAACGGTCTC |
| *INA1-REV* | TCATAGTGCTCAATACGGATAA |
| *PFK27-FOR* | CAGCTATTCAAGCGCATCT |
| *PFK27-REV* | TGGGTAAGTGTAACGGGTC |
| *NGL3-FOR* | TTTACAGCCACAAAGGAAGA |
| *NGL3-REV* | TACCGTAGCCAATCCCATA |
| *LCP5-FOR* | GCAACGACGATAGCGAAGATG |
| *LCP5-REV* | TTAGCCGTTTCCTCCACCCT |
| *PAU13-FOR* | CGGTTGAAGTTGCTGAAGCC |
| *PAU13-REV* | GTCTGGGGCAATACCAGTCA |
| *AQR1-FOR* | GCTATGTGGACGTTAATGCT |
| *AQR1-REV* | TACCTGTGAAGAAGGAACCC |
| *EKI1-FOR* | AGGCTACCACAACGACCACT |
| *EKI1-REV* | TCCATAAGGTGCGTTTCATC |
| *BAG7-FOR* | TATGAGGCTTCCAGAACTGT |
| *BAG7-REV* | ATGGTGTCCTTATTCGCTTT |
| *ERG8-FOR* | AAAACAGGGCTGGGCTCC |
| *ERG8-REV* | CGCTACATCAAACCCGCTT |
| *ECM12-FOR* | ATTTTCACATTAAGCAGCAGG |
| *ECM12-REV* | AAGGTCAAATGAAAGTCCGTC |
| *TOS3-FOR* | CTCTACTCTTACGCCTGTCGGT |
| *TOS3-REV* | CAAGTGATGCGTCTGCTTCTAT |
| *PHS1-FOR* | TTCTGCTGTTGGTTGGTCTT |
| *PHS1-REV* | TGAACAAGGGTAGCGACATT |
| *ALG5-FOR* | TCTCAAAACAGAGGCAAAGG |
| *ALG5-REV* | GAACTTACTAGCACCATCAGCA |
| *PCK1-FOR* | AGAATCCAAGGTTCCAGCAG |
| *PCK1-REV* | TAGTAGTTCGCCGATTCCTG |
| *CST26-FOR* | TCAAGAATGTGCTACTGCCTCA |
| *CST26-REV* | GCTCACCATATTCCTCCTGTTT |
| *SNZ1-FOR* | GCAGTTGGGTTGTGATGGT |
| *SNZ1-REV* | CTGGGATTGTCAAAGTGAGTG |
| *DAK2-FOR* | GCTGCTTATGATGGTGCCG |
| *DAK2-REV* | CCTTCGCTGTCGAGTTTTCT |
| *FDH1-FOR* | TCCGCTAGAGGTGCTATTTG |
| *FDH1-REV* | GGATGGTCTTTTGGTGCTG |
| *SER3-FOR* | CAACATGCGAAGAATCTGG |
| *SER3-REV* | CTCTGCTGGTAGCGTAGTC |
| *IDP3-FOR* | CCGATGAGGCAAGAATGA |
| *IDP3-REV* | AGTGAGGGACTAGACGAGGTA |
| *PCK1-FOR* | CCGCAGACCCACATAGATT |
| *PCK1-REV* | TGGCGTAACAACCACCTTC |
| *KGD1-FOR* | ATGACGTGGAAGCTGTGAC |
| *KGD1-REV* | GTTGAGTAAACGATGGCTGA |
| *CIT1-FOR* | ACTTTGAACGCAGGGAGA |
| *CIT1-REV* | TTGAGCCGTATAACGAGGA |
| *QCR9-FOR* | CTATCTTTGCAGGTGCCTTT |
| *QCR9-REV* | CGAGCCTTGACATCTTTCC |
| *POR1-FOR* | TTATTTCGCCAAAGACTACTCC |
| *POR1-REV* | GCCTTAGCACCGACCTGTA |
| *GAP1-FOR* | GCTTATCTATCCTTCCCACT |
| *GAP1-REV* | TTCTACCCGTATCAATGTCC |
| *CIN5-FOR* | GCCACGACTAACCTTCCTT |
| *CIN5-REV* | GCCTGTTTTACCGATCAACT |
| *NOP16-FOR* | AGCCAAAGTGAAAGGGAAGA |
| *NOP16-REV* | CCTCAGATTGCTGGTAGATGT |
| *DSF1-FOR* | TGTCAACGACCCAACTATCA |
| *DSF1-REV* | TCTTACCAGAGCCCATCAAAC |
| *HMS1-FOR* | ACAAGGGTGAGGACAGGAAG |
| *HMS1-REV* | TACTCGTAGCGTTGGGACA |
| *FYV7-FOR* | AGGGCAATAGAGGGAAAGAAGAA |
| *FYV7-REV* | TTGCTCGTTTCTTTGCTTGTTTA |
| *MCH2-FOR* | CTGGTATAGGAACTGCTGGAAG |
| *MCH2-REV* | CGCAATGGTGCTAAGTGATG |
| *ENA1/2/5-FOR* | CAAAGGCTATCGCTCAGGA |
| *ENA1/2/5-REV* | TAACTAAAGGTAAGACGGGCA |
| *ARI1-FOR* | ACAGTCATCGGCTCAGGTAGA |
| *ARI1-REV* | AATGGAATGGGGAGGCAGT |
| *CUP1-2-FOR* | AACGGGGTGTAACAGCGA |
| *CUP1-2-REV* | ATTTCCCAGAGCAGCATGA |
| *HIM1-FOR* | AGTGCGGAGAACTCAGGTA |
| *HIM1-REV* | AAGGCAGTTGTAACCACCA |
| *FLO11-FOR* | CCCTGTCACGACGGCTATT |
| *FLO11-REV* | TTGGCACCATTTGAACCTG |
| *COG7-FOR* | ATAATCGCAATACCAATGTGAG |
| *COG7-REV* | AACGGTCCTAACTTGTTCCAG |
| *AQY2 (m)-FOR* | ACTCCAGGCAAGGTTCTC |
| *AQY2 (m)-REV* | GCAGCCATAAAGTTAGTTTC |
| *SMC3-FOR* | ATGATAACGCTAACGACCAT |
| *SMC3-REV* | ACAGTCTTCTGACCACCTGA |
| *ACT1-2-FOR* | GCTCCAATGAACCCTAAATC |
| *ACT1-2-REV* | GGAAGAGTACAAGGACAAAACG |
